# Novel insights into the HLA class I immunopeptidome and T-cell immunosurveillance

**DOI:** 10.1186/s13073-017-0439-8

**Published:** 2017-05-24

**Authors:** Cornelis J. M. Melief, Jan H. Kessler

**Affiliations:** ISA Pharmaceuticals, J.H. Oortweg 19, 2333 CH Leiden, The Netherlands

## Abstract

Advances in mass spectrometry have allowed the high-throughput quantitative identification of human leukocyte antigen (HLA) class I ligands, and recent studies have reported on the breadth and diversity of the HLA class I immunopeptidome. These findings have far-reaching implications for immunosurveillance by T cells and translational value for immunotherapy.

## The HLA class I immunopeptidome

Human leukocyte antigen (HLA) class I molecules expose the health status of cells to CD8^+^ cytotoxic T lymphocytes (CTLs) by presenting at the surface of each cell around 10^4^ different peptide species (with varying copy numbers) processed from the cellular protein content. CTLs can eradicate cells infected with microbial pathogens and cancer cells upon recognition of pathogen-derived or tumor-specific peptides. However, CTLs also cause tissue damage in autoimmune disease if derailed T cells are reactive with self-protein-derived peptides. The T-cell receptor (TCR) mediates the specificity of recognition by binding to the HLA class-I-presented short peptide (length 8–12 amino acids), also called class I ligand, or CTL epitope when recognized by CTLs. Together, all class I ligands of a cell constitute the self class I immunopeptidome. Autoimmune responses are precluded by the deletion of autoreactive T cells before they can develop into immunocompetent T cells, occurring in the thymus as part of the central tolerance process, and by additional tolerization mechanisms of still existing autoreactive T cells in the periphery [1].

The resultant non-tolerized T-cell repertoire serves to safeguard health by scrutinizing cells for “foreign” class I ligands, derived from pathogens, tumor cells, or otherwise aberrant cells.

The mechanisms of HLA class I antigen processing, involving enzymatic degradation by the proteasome and peptidases, intracellular routing, and specific binding to the individual’s highly polymorphic HLA class I molecules, have been uncovered during the past decades. However, due to methodological constraints, in particular the dominant use of T-cell-based readouts for class I ligand detection, insights were mostly qualitative. Recently, enabled by advances in mass spectrometry (MS) instrumentation, liquid chromatography-tandem MS (LC-MS/MS) has evolved from a valuable means for qualitative identification of HLA class I ligands into a high-throughput technique for the relatively unbiased and accurate sequencing of thousands of class I ligands per analyte [2]. Important questions that require in-depth quantified profiling of the immunopeptidome can now be addressed.

## Preferential usage of sources limits the breadth of the HLA class I immunopeptidome

HLA class I ligands predominantly originate from rapidly degraded proteins or defective translation products [3], but it has been unclear whether specific features of proteins exist that render them more prone to becoming class I ligands.

Pearson and colleagues set out to investigate this with a proteogenomic approach [4]. Using MS/MS sequencing, they first identified 25,270 non-redundant class I ligands eluted from immortalized B cell lines (B-LCLs) of 18 individuals expressing in total 27 different prevalent HLA class I molecules. Remarkably, the ligands originated from only 6195 out of the 10,575 gene products expressed in these B-LCLs, so that 41% of expressed genes in the B-LCLs did not give rise to a class I ligand. The authors then searched for the features that distinguish the source from non-source genes. RNA transcript level, the related protein abundance, and protein length were strongly associated with source genes, constituting factors expected both intuitively and from results of previous studies [5]. In addition, novel characteristics were identified: (1) class I ligand source transcripts were enriched in features contributing to translation efficiency; (2) the secondary structure of source proteins showed a bias towards more sheet motifs; (3) motifs that serve as proteolytic signal and lysine ubiquitination sites also occurred in higher frequency in source proteins and they were more disordered, favoring proteasomal degradation; and (4) gene ontology analyses confirmed previous findings that class I ligand source proteins more often interact with RNA, DNA, or other proteins, whereas cell membrane proteins were overrepresented in the non-source set [4].

This study thus defines features underlying and explaining the distinction between source and non-source transcripts and proteins. A logistic regression model based on these features predicted with accuracy whether a gene product could generate class I ligands. This has translational importance for immunotherapy (discussed below). The selective usage of gene products limits the breadth of the self class I immunopeptidome, defined as the proportion of the proteome giving rise to ligands, and thus CTL tolerization against peptides from non-source self proteins may be completely absent, which also has translational significance.

## A special proteasomal mechanism enhances the diversity of the HLA class I immunopeptidome

Another important remaining issue concerned not the sources, but the way that class I ligands are generated from these sources. Canonical HLA class I ligands are liberated during a first digestive step from their source in the channel of the complex multi-subunit and multi-catalytic proteasome [6]. Proteasomal degradation has a stochastic nature but follows rules allowing its predictive modeling. Deviating from normal class I ligand generation, previous anecdotal reports demonstrated a remarkable additional activity of the proteasome, namely the ligation of two peptides that are first generated, leading to a CTL epitope [6].

Additional CTL epitopes were subsequently also shown to arise by proteasome-catalyzed peptide splicing (PCPS), as the process was named, as proven by the fact that they are composed of two amino acid sequences from non-contiguous areas of a protein joined together in the proteasome [6]. All these studies depended on T cells, so PCPS-derived ligands could not be systematically searched.

Liepe and co-workers [7] have now exploited the power of MS/MS-based ligand identification in conjunction with newly devised proteome database search strategies to chart the proportion of PCPS-dependent class I ligands among over 10,000 identified. They report that, in different cell types, as much as 21–32% of the peptides eluted from HLA class I molecules are spliced sequences, which are on average expressed with a slightly lower abundance than non-spliced class I ligands, thus accounting for approximately one-quarter of all class I presented peptides. Therefore, this study [7] firmly establishes that PCPS is not a rare trick of the proteasome, as was suggested previously [6], but an important function.

**Fig. 1:**
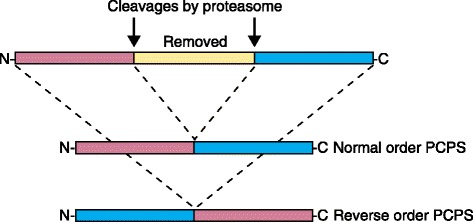
Spliced peptides presented in HLA class I molecules are mainly formed in two ways. After appropriate cleavage by the catalytic subunits of the proteasome the two splice reactants derived from one parental peptide (*cis*-splicing) can be ligated either in the normal order (*top variant*) or in the inverted reverse order of the parental amino acid sequence (*bottom variant*). The intervening sequence is removed, giving rise to a novel antigenic sequence if the novel peptide has appropriate HLA class I binding capacity

PCPS was already known to rely on a transpeptidation reaction [8], and it can occur in the normal (natural) amino acid order of the parental protein or in the inverted reverse order [6]; Liepe and colleagues [7] showed that these variants occur at similar frequencies (Fig. 1). Only *cis-*splicing of degradation fragments from one source protein has been observed in cells, while *trans-*splicing of peptides from different sources, being necessarily much more random, was not observed [7].

With the large pool of spliced ligands available, Liepe et al. [7] also studied the length of the excised intervening sequence, which appeared to vary between 1 and 20 amino acids, and searched for possible sequence preferences dictating PCPS. Although PCPS sequence rules were suggested previously from cell-free experiments [9], the results from the study by Liepe et al. [7] indicate that distinctive rules are hard to deduce, and consequently modeling for reliable prediction of PCPS-derived ligands is not possible at present and may be a bridge too far even when more training data become available. An intriguing finding was that the PCPS-dependent ligands do not completely abide to the known class I peptide-binding motifs, a phenomenon that awaits further research. The significant freedom in the ligation mechanism implies that PCPS tremendously enhances the immunopeptidome’s diversity (defined as the number of different ligands originating from a protein). Many proteins were only represented as spliced class I ligands [7]; thus, immunosurveillance against these proteins depends solely on peptide splicing.

## Implications for predicting the immunopeptidome and translational relevance

Selective steps in class I ligand generation, in particular class I peptide binding, have been modeled, enabling in silico prediction [10]. Separately or taken together these algorithms are widely used for the prediction of CTL epitopes in viral or tumor-associated proteins for the monitoring and design of immunotherapeutic strategies, in particular vaccination. As the power of the prediction tools is at present not accurate enough, experimental validation of epitopes is mostly performed.

Neo-CTL epitopes that arise from personal tumor-specific somatic mutations, mostly in self proteins, have recently been identified as ideal targets for cancer immunotherapy [11]. Design of a personalized cancer vaccine disqualifies lengthy experimental epitope validation, increasing the need for improved predictions. Here the results of Pearson et al. [4] come in handy, because their predictive modeling of source features adds a non-redundant level to CTL epitope prioritization. As class I cancer immunopeptidomes are increasingly analyzed on a large scale [11], such predictive modeling may further improve. Furthermore, the identification of self class I immunopeptidomes and their prediction based on source features enables deprioritization of any predicted vaccine CTL epitope in which the TCR-binding residues share too much homology with those of putative self class I ligands (with the same class I restriction, that is, binding only to the same HLA molecule), thus preventing immunizations with non-immunogenic class I ligands (because of T-cell deletion) or potential triggering of (still existent) autoreactive T cells.

The findings of Liepe and colleagues [7] might not help in “predictive” vaccine design, but underline the importance of direct identification of immunopeptidomes using sophisticated database search strategies and reveal the potential importance of PCPS-generated CTL epitopes in any disease-related T-cell response. With respect to rational design of neoantigen-directed personal cancer vaccines, further optimization of MS techniques would enable us to incorporate PCPS-type epitopes into such approaches on a routine basis. The novel insights into the intracellular selection of protein sources for class I ligands and the mechanisms involved in their generation together contribute to a better understanding of CTL immunosurveillance and help to improve the immunomonitoring and predictive design of immunotherapy for cancer.

## Abbreviations

B-LCLs: Immortalized B cell lines
CTL: Cytotoxic T lymphocyte
HLA: Human leukocyte antigen
LC-MS/MS: Liquid chromatography-tandem mass spectrometry
MS: Mass spectrometry
PCPS: Proteasome-catalyzed peptide splicing
TCR: T-cell receptor
